# Survey highlighting the lack of consensus on diagnosis and treatment of patent ductus arteriosus in prematurity

**DOI:** 10.1007/s00431-022-04441-8

**Published:** 2022-03-19

**Authors:** Tim Hundscheid, Afif El-Khuffash, Patrick J. McNamara, Willem P. de Boode

**Affiliations:** 1 Department of Pediatrics, Division of Neonatology, Radboud Institute for Health Sciences, Amalia Children’s Hospital, Radboud University Medical Center Nijmegen, Internal Postal Code 804, Geert Grooteplein Zuid 10, 6525 GA Nijmegen, The Netherlands; 2 Department of Neonatology, The Royal College of Surgeons in Ireland, Dublin, Ireland; 3 Departments of Pediatrics & Internal Medicine, Division of Neonatology, University of Iowa, Iowa City, IA USA

**Keywords:** PDA, Ibuprofen, Placebo, Morbidity, Mortality, Conservative approach

## Abstract

To gain insight in the availability of guidelines, diagnostic criteria, and treatment strategies and whether clinical equipoise regarding optimal treatment for patent ductus arteriosus (PDA) in prematurity is present. We hypothesized that (co-)authors of PDA-related papers were more likely to screen for a PDA and would treat earlier and more aggressively. An international internet-based survey between September 2019 and March 2020 in which we collected (1) baseline characteristics; (2) availability of guidelines; (3) screening strategy for PDA; (4) diagnostic criteria for hemodynamic significance; (5) treatment strategy; and (6) metrics of treatment efficacy. Finally, ten clinical equipoise statements were posed on a Likert scale. In total, 144 surveys were sent, of which 71/144 (49%) surveys could be analyzed with 56/71 (79%) fully completed surveys. The respondents, mainly neonatologists in a level III neonatal intensive care unit, of whom 36/71 (51%) had (co-)authored a publication on the PDA, highlighted a lack of national guidelines, heterogeneous approach to screening strategies, and marked variability in diagnostic criteria to assess hemodynamic significance, treatment strategies and effect measurement. No major significant differences were observed between respondents who did or did not (co-)author a publication on the PDA. Respondents who screened for PDA scored significantly higher on the need for screening, early and aggressive treatment. Remarkably, the scores of all statements regarding clinical equipoise varied widely.

*   Conclusions*: Our survey highlights the lack of guidelines and enormous heterogeneity in current practice. Current evidence is not robust enough to harmonize current treatment strategies into (inter)national guidelines.

**Table:** 

**What is Known:** *• Patent ductus arteriosus (PDA) incidence is inversely related to gestational age.* *• Although early pharmacological treatment induces PDA closure, optimal treatment is debated due to the lack of beneficial effects on outcome.*	
**What is New:** *• In the absence of (inter)national guidelines, diagnostic and treatment strategies are heterogeneous and contradictory, even in a selected hemodynamically- interested group.* *• Different PDA screening strategies did, while PDA publication status did not, show significant differences in treatment strategy and responses to equipoise statements.*

**Supplementary information:**

The online version contains supplementary material available at 10.1007/s00431-022-04441-8.

## Introduction

Management of patent ductus arteriosus (PDA) in preterm infants remains controversial. Current evidence from randomized controlled trials (RCTs) and meta-analyses shows no clear beneficial effect of pharmacological treatment over placebo regarding mortality or neonatal morbidity [1]. Many clinicians prefer to treat pharmacologically, although the methodological quality of the conducted studies is moderate to low; specifically, most trials end up representing a comparative evaluation of early versus later pharmacological treatment since on average over 50% of patients in control group received open-label treatment during the study [2, 3]. On the contrary, other centers have adopted a conservative approach (watchful waiting) [4]. Parallel to this clinical divergence towards PDA management, recent trials addressed both conservative management and combined pharmacological treatment (i.e., paracetamol in addition to ibuprofen) [5, 6].

One of the main limitations for selective pharmacological treatment is the lack of robust diagnostic criteria to define hemodynamic significance [7]. Of concern, the diagnostic criteria used in PDA RCTs represent either a dichotomous assignment as PDA present/persistent or use limited echocardiography criteria with poor reproducibility [8]. Nevertheless, recent evidence suggests that PDA severity scores based on comprehensive echocardiographic criteria may enable identification of higher-risk patients at greater risk of abnormal neonatal outcomes [9, 10].

To gain more insight in current practices regarding the diagnosis and management of persistent PDA in preterm infants less than 28 weeks’ gestation, we conducted an international internet-based survey. We focused on the availability of (national) guidelines, screening protocols, diagnostic criteria, treatment strategies, and definitions of treatment effect. Moreover, we analyzed the response on several (controversial) statements with respect to PDA management. We hypothesized that (co-)authors of PDA-related papers were more likely to screen for a PDA and would treat earlier and more aggressively.

## Materials and methods

A comprehensive internet-based survey addressing these current controversies in PDA assessment and management was originally designed by two authors (TH and WdB) based on the available literature. The survey was tested and then reviewed and edited by the other authors (AK and PM) (Supplement 1). Via the Castor^®^ Database, a survey invitation was sent to all members of the Section Circulation, Oxygen Transport, and Hematology of the European Society of Pediatric Research (ESPR) and principal investigators of the BeNeDuctus trial between September 2019 and March 2020 [2, 11]. A reminder was sent twice, according to published recommendations [12]. Additionally, a QR-link to apply for the questionnaire was shared within the ESPR newsletter and invited section members were asked to share this in their network. After application, an invitation to the survey was sent to applicants by the author (TH). The used Checklist for Reporting Results of Internet E-Surveys (CHERRIES) is presented in Supplement 2 [13].

The following information was collected: (1) baseline characteristics of the respondent and the institution; (2) availability of both local and national guidelines; (3) screening strategy for PDA; (4) diagnostic criteria used to determine hemodynamic significance; (5) treatment strategy; and (6) metrics of treatment efficacy. At the end of the survey, ten (controversial) statements addressing clinical equipoise were posed on Likert scales which respondents were asked to rank.

Data were collected and analyzed anonymously. All responses with reported baseline characteristics were included. We were interested in differences between respondents who did or did not (co-)author at least one publication on the PDA, as a proxy for relevant PDA research involvement. Furthermore, we looked at differences between respondents who did and did not routinely perform echocardiographic screening for PDA. Statistical analyses were performed using descriptive statistics, presented as median (interquartile range) and number/total respondents, percentage. The Mann–Whitney *U* test was used for comparison between two independent groups.

## Results

In total, 144 surveys were sent (95 ESPR section members, 12 BeNeDuctus trial principal investigators, and 37 applicants) of which 56 respondents completed the survey in full (39%). Another 15 (10%) respondent surveys had completed baseline characteristics and at least one subgroup and therefore were also included. All answers are summarized in Supplement 3.

### Baseline characteristics

Most respondents were neonatologists (65/71, 92%), with 10–20 years’ experience (30/71, 42%) working in a level III (23/71, 32%) or IV (45/71, 63%) neonatal care unit [14]. Respondents originated from 25 different countries (Fig. S1 and Table S1). There was an overrepresentation of respondents from The Netherlands and no respondents from Asia. In most respondents’ centers, a pediatric cardiology service was available 24/7 (52/71, 73%) and surgical ligation was performed on site (49/71, 69%). Half of the respondents (36/71, 51%) reported to have (co-)authored a publication on the PDA in preterm infants in peer-reviewed medical journals (Table S1).

### Guidelines

Only 4/70 respondents (6%), which equates to 16% of countries (i.e., The Netherlands, Germany, Turkey, and Poland), indicated that a national guideline regarding diagnosis and management of PDA in preterm infants (gestational age < 28 weeks) was available. Interestingly, other respondents from three of these countries (i.e., The Netherlands, Germany, and Turkey) stated that no national guideline existed. Local guidelines were available according to 40/71 respondents (56%). No local guideline was made without involvement of a (hemodynamic consultant) neonatologist (Table S2).

### Screening strategy

Routine screening of preterm infants for PDA was reported by 33/71 (46%) participants. Most respondents (20/33, 61%) performed echocardiographic screening between 24 and 72 h postnatal age (Table S3). Criteria for screening were based on gestational age alone (14/31, 45%), or gestational age in combination with birth weight (17/31, 55%) (Table S3). Echocardiographic evaluation of patients in centers where routine screening is not performed was mainly based on clinical signs (36/38) and/or additional assessments (16/38) (Table 1). Only 15/71 respondents (21%) would perform routine echocardiographic PDA screen prior to administration of a second dose of surfactant. If a large left-to-right transductal shunt was identified, most (13/15, 87%) would not administer a second dose of surfactant (Table S3).

**Table 1:** Reasons to perform echocardiography for respondents without a ductal screening program (*n* = 38)

Clinical signs	36	Additional assessments	16
Heart murmur	33	Chest radiograph	10
Increased FiO2	32	Blood lactate	10
Low diastolic arterial pressure	29	Cerebral US with absent/reversed diastolic blood flow	9
Wide pulse pressure	28	Signs of renal failure	7
Inotropic support	28	NIRS monitoring	6
Tachypnea / pulmonary edema	28	NT pro BNP	5
Ventilator dependency	28		
Bounding pulses	25		
Hyperactive precordium	25		
Low mean arterial pressure	24		
Extubation failure	20		
CPAP failure	15		
Renal impairment	10		
Feeding intolerance	9		

Data are presented as number

*BNP* brain natriuretic peptide, *CPAP* continuous positive airway pressure, *FiO2* oxygen requirement, *NIRS* near-infrared spectroscopy, *US* ultrasound

### Diagnostic criteria for (hs)PDA

**Fig. 1:**
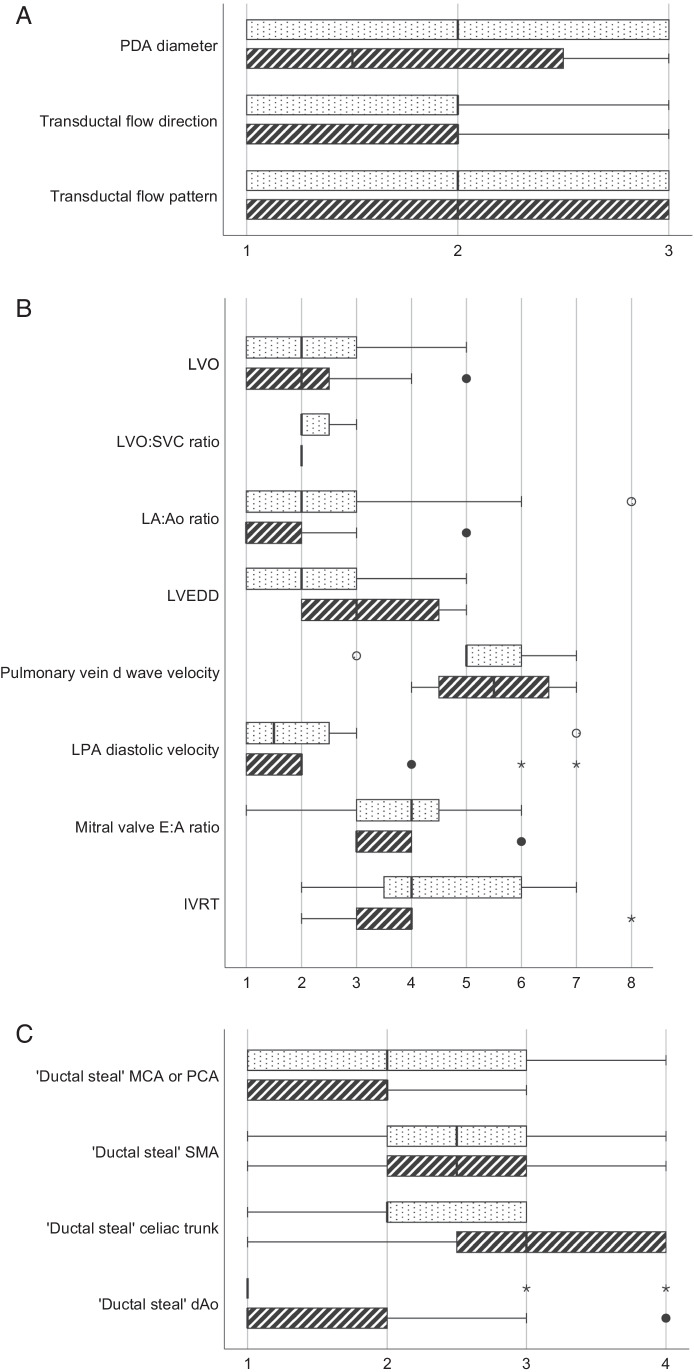
Ranking PDA characteristics. Footnote: Data is presented as boxplots with median, interquartile ranges, and minimum/maximum ranking (*x*-axis), for participants who did (dotted bars) and did not (dashed bars) publish on the PDA. All echocardiographic parameters are subdivided in (**a**) PDA characteristics (rank 1–3); (**b**) indices of pulmonary overflow (rank 1–8); and (**c**) indices of systemic hypoperfusion (rank 1–4). Statistical difference (*p* < 0.05) for “ductal steal” celiac trunk for PDA publication status. *dAo*, descending aorta; *IVRT*, isovolumic relaxation time; *LA:Ao*, left atrium:aorta; *LPA*, left pulmonary artery; *LVEDD*, left ventricular end-diastolic dimension; *LVO*, left ventricular output; *MCA*, middle cerebral artery; *PCA*, pericallosal artery; *PDA*, patent ductus arteriosus; *SMA*, superior mesenteric artery; *SVC*, superior vena cava

Although echocardiographic assessment is routinely performed by similar rates of neonatologists with hemodynamics expertise and pediatric cardiologists, the first patient assessment where exclusion of congenital heart disease is paramount is mostly performed by the latter (40/71, 56%). If the first patient assessment was performed by a neonatologist, it was frequently reviewed by a pediatric cardiologist in a timely manner (19/71, 27%) (Table S4). Parameters used to define hemodynamic significant PDA (hsPDA) varied widely. Most respondents (49/59, 83%) did not use any form of a PDA severity score [9, 15]. Reported cut-off values for echocardiographic parameters are presented in Table 2. The ranked importance of echocardiographic parameters for the determination of a hsPDA is shown in Fig. 1. Respondents that published on the PDA gave a higher ranking for abnormal OR retrograde diastolic flow (“ductal steal”) in the celiac trunk.

**Table 2:** Echocardiographic parameters with cut-off value for small and large shunts

		*n*	Cut-off value small shunt	Cut-off value large shunt
PDA diameter	(mm)	41	< 1.5 (1.5–1.5)	> 2.0 (1.8–2.3)
	(mm/kg)	9	< 1.4 (1.0–1.5)	> 1.5 (1.5–2.0)
	PDA:LPA	5	< 1.0 (0.5–1.0)	> 1.1 (1.0–1.8)
LA:Ao ratio		38	< 1.5 (1.4–1.5)	> 1.5 (1.5–2.0)
Transductal flow velocity (v_max_) (m/s)		19	> 2.0 (2.0–2.5)	< 2.0 (1.5–2.0)
LVO (ml/kg/min)		22	< 250 (200–300)	> 300 (300–400)
LPA diastolic velocity (m/s)		18	< 0.3 (0.2–0.3)	> 0.4 (0.2–0.5)
LVEDD (mm)		10	< 12.0 (10.0–12.5)	> 15.0 (12.8–18.0)
Mitral valve E:A ratio		15	< 1.0 (1.0–1.0)	> 1.0 (1.0–1.0)
IVRT (ms)		12	> 40 (40–55)	< 35 (30–45)
LVO:SVC ratio		2	< 4.0 (4.0–4.0)	> 4.0 (4.0–4.0)
Pulmonary vein d wave velocity (m/s)		4	< 0.3 (0.2–0.5)	> 0.5 (0.5–0.6)
		Small shunt	Moderate shunt	Large shunt
Transductal flow pattern (*n* = 37)	Growing	8 (22)	18 (49)	11 (30)
	Pulsatile (non-restrictive)	0	10 (27)	27 (73)
	Restrictive	32 (86)	0	5 (14)

Data are presented as number (percentage) with median (interquartile range) for cut-off values. Percentages may not sum to 100 due to rounding

*IVRT* isovolumic relaxation time, *LA:Ao* left atrium:aorta, *LPA* left pulmonary artery, *LVEDD* left ventricular end-diastolic dimension, *LVO* left ventricular output, *SVC* superior vena cava, *v*_*max*_ maximum velocity

### Treatment strategy

The most commonly used treatment strategies were early targeted treatment based on screening at a postnatal age of 24–72 h (26/61, 43%) and symptomatic treatment at a postnatal age > 72 h (25/61, 41%). In most centers, the neonatologist decides to start treatment (35/59, 59%) (Table S5). Early targeted treatment was significantly more often used as treatment strategy by respondents who routinely screen echocardiographically (20/29, 69%) in comparison to those who did not (6/32, 19%).

Most respondents reported pharmacological treatment with ibuprofen (43/57, 75%). Indomethacin (6/57, 11%) and paracetamol (8/57, 14%) were less commonly prescribed in the countries surveyed. Most respondents (37/43, 86%) start with a median loading dose of 10.0 (10.0–10.0) mg/kg ibuprofen and subsequent median doses of 5.0 (5.0–5.0) mg/kg (Table 3).

**Table 3:** Used drug(s) and dosage for first and second course

		First course	Second course
**What is the drug of first choice in your center?**			
Ibuprofen		43 (75)	33 (67)
Indomethacin		6 (11)	7 (14)
Paracetamol/acetaminophen		8 (14)	9 (18)
**Do you start with a loading dose?**			
* Ibuprofen*		43	32
Yes		37 (86)	26 (81)
	*Loading dose* (*mg/kg*)	10.0 (10.0–10.0) 5.0 (5.0–5.0)	10.0 (10.0–10.0) 5.0 (5.0–5.0)
	*Subsequent dose(s)* (*mg/kg*)		
No		6 (14)	6 (19)
	*Dosage* (*mg/kg*)	15.0 (10.0–20.0)	7.3 (5.5–9.4)
* Indomethacin*		6	7
Yes		4 (67)	1 (14)
	*Loading dose* (*mg/kg*)	0.2 (0.2–0.2) 0.1 (0.10–0.18)	0.2 0.1
	*Subsequent dose(s)* (*mg/kg*)		
No		2 (33)	6 (86)
	*Dosage* (*mg/kg*)	0.2 (0.2–0.2)	0.2 (0.18–0.23)
* Paracetamol/acetaminophen*		8	9
No		8 (100)	9 (100)
	*Dosage* (*mg/kg*)	15.0 (15.0–15.0)	15.0 (15.0–15.0)
**What is the preferred route of administration?**			
* Ibuprofen*		43	32
Per os		11 (26)	8 (25)
Intravenously		32 (74)	24 (75)
* Indomethacin*		6	7
Intravenously		6 (100)	7 (100)
* Paracetamol/acetaminophen*		8	9
Per os		2 (25)	5 (56)
Intravenously		6 (75)	4 (44)
**What is the total number of doses?**			
* Ibuprofen*	3 doses	40 (100)	31 (97)
	5 doses	-	1 (3)
* Indomethacin*	3 doses	5 (83)	7 (100)
	6 doses	1 (17)	-
* Paracetamol/acetaminophen*	12 doses	7 (88)	6 (67)
	20 doses	-	1 (11)
	28 doses	1 (13)	2 (22)

Data are presented as number (percentage) with median (interquartile range) for dosages. Percentages may not sum to 100 due to rounding

A minority (6/58, 10%) of respondents reported that surgical ductal ligation was never performed. In addition, most respondents (39/58, 67%) reported using stricter criteria for PDA ligation. Transcatheter closure was considered an option for patients with PDA by half (28/54, 52%) of the respondents. Feeding strategies during pharmacological treatment varied widely from normal advance of enteral feeding (31/52, 60%) to no increase (15/52, 29%) or even discontinuation of enteral feeding (6/52, 12%) (Table S5).

### Treatment efficacy metrics

The timing of treatment effect measurement varied as did the definition of treatment success (Table S6). Most respondents performed echocardiography after a full course (43/59, 73%). Treatment success was most commonly (25/58, 43%) defined as a change from “hsPDA to non-hsPDA,” rather than PDA closure (8/58, 14%). Undocumented PDA closure was followed up in the outpatient clinic with echocardiography by most of the respondents (41/59, 69%). As our survey mainly focused on preterm infants born at gestational age < 28 weeks, we asked respondents if there would have been differences in their response if the focus of the survey was extreme preterm infants born at gestational age < 24 weeks only; interestingly, 27/59 (46%) respondents indicated that there would be a difference with a tendency towards more aggressive treatment.

### Clinical equipoise statements

**Fig. 2:**
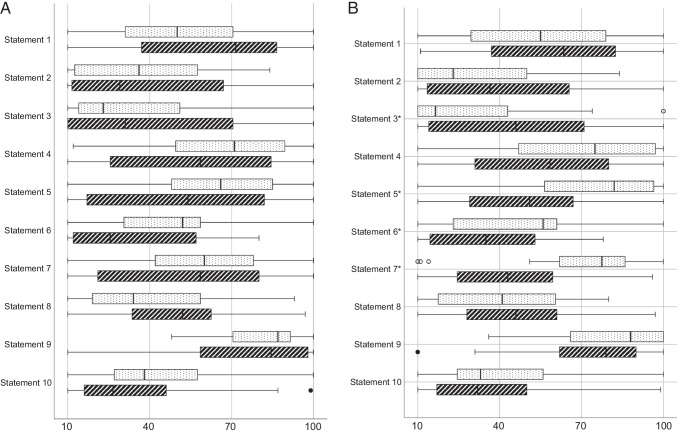
Clinical equipoise statements. Footnote: Data is presented as boxplots, with median, interquartile range, and minimum/maximum on a 10–100-Likert scale (*x*-axis) for all statements for (**a**) participants who did (dotted bars) and did not (dashed bars) (co-)authored a publication on the PDA; and (**b**) participants who did (dotted bars) and did not (dashed bars) screen for PDA. *p < 0.05. (1) PDA should be considered as an epiphenomenon of prematurity *(an indicator of immaturity)* rather than a leading cause of mortality and morbidity; (2) … therefore, it should not be treated at all; (3) … therefore, screening for ductal patency is not indicated at all; (4) PDA should be considered as an important cause of mortality and morbidity in preterm infants; (5) … therefore, screening for ductal patency is essential; (6) … therefore, it should be treated aggressively; (7) … therefore, it should be treated early; (8) The PDA diameter is a good surrogate for shunt volume; (9) I would consider treating a PDA in case of “hemodynamic significance” (any definition); (10) I would consider treating a “non-hemodynamic significant” (any definition) PDA in case of associated clinical findings/morbidity

As shown in Fig. 2a, b, almost all answers on the statements varied widely, ranging from totally disagree (10) to totally agree (100). There were no significant differences between participants who did versus those who did not publish on the PDA (Fig. 2a). Contrarily, significant differences were found between participants who did and those who did not routinely screen for PDA (Fig. 2b). Participants who routinely screened for PDA had significantly higher Likert scale scores on the statements regarding the need for screening and the need to treat early and aggressively than those who did not routinely screen.

## Discussion

After more than 40 years of clinical research, including many RCTs, PDA management in preterm infants remains a subject of great controversy and there is still no consensus on *whether*, *when*, and *how* to treat a PDA in preterm infants. This is further highlighted by our survey. Of interest, a previous survey found important differences in the perception of the pathophysiological role of PDA in neonatal morbidity and mortality between neonatologists and pediatric cardiologists [16]. Neonatologists were more likely to recommend watchful waiting, while pediatric cardiologists preferred intervention. This survey was, however, mainly answered by neonatologists; therefore, it was not possible to analyze differences between professional groups. We did, however, compare perspectives of neonatologists with hemodynamics expertise using PDA publication track record as a surrogate of expertise. Neonatologists who have published on the PDA rank the importance of “ductal steal” in celiac trunk higher. We observed a lack of the availability of national guidelines, which highlights the paucity of robust evidence favoring one specific diagnostic or therapeutic regimen. Interestingly, in Germany, most respondents (3/4, 75%) were unaware of their available, although expired, national guideline (https://gnpi.de/leitlinien/), while in The Netherlands, 1/15 (7%) stated that a national guideline was available, although there was none (personal communication). Local guidelines were more commonly available, which were mainly initiated by the neonatologists.

Ibuprofen was the most reported treatment among respondents, both for a first and second course. Optimal ibuprofen dosage and route of administration are still debated [1], which was highlighted in our survey by heterogeneous regimens of ibuprofen. Although most studies on indomethacin investigate 0.2 mg/kg loading dose, followed by 0.1 mg/kg, one-third of the respondents used a higher dosage [17]. As both ibuprofen and indomethacin are associated with adverse events, paracetamol is gaining interest as alternative treatment [18]. Although off-label for PDA treatment, paracetamol was used as first-line treatment by 14% of our respondents. There is conflicting evidence regarding the safety of the use of paracetamol in preterm infants for ductal closure [19–22]. Furthermore, superiority over conservative management has not been proven for paracetamol [5, 23]. A survey in the UK showed that ibuprofen was the first-line treatment in most departments (92%) [24]. Although 33% of respondents used paracetamol in addition, great variance in dosage, duration of treatment, and monitoring were noted. In New Zealand and Australia, paracetamol was mentioned in half of the available PDA treatment protocols and 70% of participants prescribed paracetamol [18]. With the available evidence, in our opinion, paracetamol should not be used as first-line treatment and might only be considered in case of contraindications for ibuprofen and indomethacin.

The latest American Academy of Pediatrics statement states that early routine treatment to induce PDA closure in the first 14 days of life does not improve long-term outcomes [25]. Half of the respondents perform echocardiographic screening to actively search for an asymptomatic, but hemodynamic significant, PDA, and subsequentially start early targeted treatment rather than routine treatment. However, this strategy has also not been associated with improved short-term outcomes [26]. Echocardiographic parameters used to determine hsPDA varied as did threshold values for either a small or a large shunt. The median cut-off value is grossly within the range of previously published cut-off values [27]. Neonatologists who have published on PDA ranked flow reversal in the post-ductal aorta highest as a marker of hemodynamic significance. This is an interesting observation and aligns with MRI data which concluded strongest correlation with PDA shunt volume [28]. Echocardiographic follow-up, to appraise treatment efficacy, is mainly performed after completion of a full course of treatment. This is noteworthy in the face of published evidence that repeated echocardiography after each dose of indomethacin or ibuprofen offers dose minimization and potential avoidance of adverse treatment effects [29–31]. Despite the lack of consensus on the definition of a hsPDA [7], most respondents indicated the change from a hsPDA to a non-hsPDA as definition of treatment success, rather than complete elimination of PDA flow (PDA closure).

A minority of respondents would perform echocardiographic screening for a PDA before a second dose of surfactant. Most of those who did would not give a second dose of surfactant in case of a large left-to-right shunt. Although the evidence to support this restrictive surfactant replacement strategy is scarce [32], from a pathophysiological point of view, surfactant could further decrease the pulmonary vascular resistance, thereby increasing the ductal blood flow to the lungs, ultimately increasing the risk of pulmonary hemorrhage [33]. There might be a selection bias in this question, as people who deliberately perform a scan prior to the second surfactant dose will be more likely to withhold the second dose in case a large PDA is found.

Interestingly, the contradicting statements “*PDA should be considered as an epiphenomenon of prematurity (an indicator of immaturity) rather than a leading cause of mortality and morbidity*” and “*PDA should be considered as an important cause of mortality and morbidity in preterm infants*” were scored equally. On the contrary, there was low responder agreement on a “no screening and/or treatment” policy and higher agreement on “active screening with subsequent early and aggressive treatment.” The discordance between opinions on PDA relevance and approach to screening highlights the confusion among clinicians. No statistical differences were found between participants who did and those who did not publish on the PDA. Respondents that do screen in comparison to those who do not screen for a PDA started treatment earlier. Regarding the clinical equipoise statements, they scored much higher on early screening and aggressive treatment.

The statement with the highest respondent score was “*I would consider treating a PDA in case of ‘hemodynamic significance’ (any definition)*.” Although there seems to be agreement on the fact that PDA treatment should be considered when there is evidence of hsPDA, robust diagnostic criteria are lacking. This was highlighted by the heterogeneity on the definition of hsPDA in our survey. This suggests the need to standardize (echocardiographic) assessment of hsPDA and aim to include only patients with a well-defined hsPDA in future RCTs. In conclusion, the responses to statements demonstrate marked heterogeneity in the opinion about optimal diagnostic and therapeutic management strategy in preterm infants with a persistent PDA. This perspective was also observed in a recent RCTs, in which lack of physician equipoise led to the exclusion of 21% of eligible patients, many of whom were the sickest and most immature patients [23]. In order to minimize open-label treatment, as was successful in a recent RCTs [34], physicians at participating hospitals must retain clinical equipoise in the pursuit of answering the question whether or not a PDA in vulnerable preterm infants is an innocent bystander or the prime suspect.

One of the main limitations of our study is that it was conducted mainly in a special interest group, which might threaten the external validity and generalizability of our findings. In an attempt to reduce selection bias, the principal investigators on the BeNeDuctus trial were also invited and participants could apply for the survey via a QR code which was shared widely. In fact, our findings might even be more concerning as it highlights that even in a special interest group, there is limited consensus in any aspect of PDA diagnosis and management. This was further highlighted by the absence of major differences between participants who did (co-)author a publication on the PDA and those who did not. It is not clear whether a track record of PDA publication reflects expertise in PDA physiology or echocardiography both of which may impact perspectives. The number of respondents that was funded to perform research on the PDA was too little to perform subgroup analyses (16/71 (23%)). Another limitation is the relatively low response rate, which might be due to the length of this comprehensive survey.

Current RCTs, like the Baby-OSCAR trial [35], management of the PDA trial (NCT03456336), and the BeNeDuctus trial [36], will hopefully add to the available knowledge and will guide our way either to more aggressive treatment with monotherapy (ibuprofen or paracetamol) or even combined therapy (ibuprofen and paracetamol) or to a more expectant approach [37, 38]. Furthermore, advances in percutaneous ductal closure might lead to reconsideration of early invasive closure for a select group of patients [39]. Unfortunately, inclusion criteria and definitions of (hs)PDA remain heterogeneous, as do treatment strategies. Therefore, meta-analysis of currently available RCTs will not be likely to answer the question whether or not a (hs)PDA should be treated. We suggest a consensus statement meeting to define a hsPDA which then could be uniformly used as an inclusion criterion in future RCTs.

In conclusion, this international survey demonstrates that current evidence regarding PDA diagnosis and management is not robust enough to support the development of (inter)national guidelines. It shows enormous heterogeneity in screening strategies, diagnostic criteria for hsPDA, treatment strategies, and effect measurements. The current lack of clinical equipoise was further highlighted by the wide variance in responses on standardized statements regarding management of a persistent PDA in preterm infants, which is a threat for recruiting RCTs.

## Supplementary information

Below is the link to the electronic supplementary material.

Supplementary file1 (DOCX 24 KB)

Supplementary file2 (DOCX 20 KB)

Supplementary file3 (DOCX 28 KB)

Supplementary file4 (PNG 836 KB)

## Data Availability

The database with survey results will be available upon reasonable request.

## Abbreviations

ESPR: European Society of Pediatric Research
(hs)PDA: (Hemodynamic significant) Patent ductus arteriosus
PDA: Patent ductus arteriosus
RCT: Randomized controlled trials
