# Supplementary material for: Survey highlighting the lack of consensus on diagnosis and treatment of patent ductus arteriosus in prematurity
Source: Eur J Pediatr. 2022 Mar 19;181(6):2459–68. doi: 10.1007/s00431-022-04441-8 (PMC9110525; doi:10.1007/s00431-022-04441-8)
Supplement: Supplementary file 1 — Supplementary file1 (DOCX 24 KB) [file 431_2022_4441_MOESM1_ESM.docx]

**Supplement 1 –** PDA survey

## Baseline characteristics

- 1. I am a
- Neonatologist
- Neonatal fellow
- Pediatric cardiologist
- Other, namely *(please specify)*
  1. I have been working on a neonatal intensive care unit for
- < 10 years
- 10-20 years
- > 20 years
  1. I am currently working in *(country)*
  2. I work in a, as defined by American Academy of Pediatrics Committee on Fetus And Newborn[14]
  - Level I neonatal care unit (‘Special Care Baby Units’(SCBU). Babies who need more care than healthy newborns but are relatively stable and mature.)
  - Level II neonatal care unit (‘Local Neonatal Units’, ‘Special Care’, ‘High Dependency’ (HDU), these can look after babies who need more advanced support.)
  - Level III neonatal care unit (‘Neonatal Intensive Care Units’ (NICU).
  - Level IV neonatal care unit (‘Surgical NICU’)
  1. Have you been funded to investigate the PDA?
- Yes
- No
  1. Have you published on the PDA?
- Yes
- No
  - 1. If yes, how many publications regarding PDA do you (co-)authored?
- 1-5
- 5-10
- > 10
  1. Yearly admissions of preterm infants (24-28 weeks)
  - < 25 preterm infants 24-28 weeks
  - 25-50 preterm infants 24-28 weeks
  - 50-100 preterm infants 24-28 weeks
  - > 100 preterm infants 24-28 weeks
  1. Yearly admissions of extreme preterm infants < 24 weeks
  - < 10 extreme preterm infants < 24 weeks
  - 10-25 extreme preterm infants < 24 weeks
  - 25-50 extreme preterm infants < 24 weeks
  - > 50 extreme preterm infants < 24 weeks
  1. Is there a pediatric cardiology service available at your institution?
- No
- No, only remote consultation
- Yes, but only during daytime
- Yes, 24/7
  1. Do patients have to be transferred to another center for surgical ductal ligation?
  - Yes
  - No
    1. If no, where is surgical ductal ligation (mostly) performed?
- In the NICU
- In the operating room (OR)
- Cath lab

## Guidelines

- 1. Is there a national guideline available regarding the diagnosis and management of persistent PDA in preterm infants (< 28 weeks)?
- Yes
- No
  1. Is there a local guideline available at your institution regarding the diagnosis and management of persistent PDA in preterm infants (< 28 weeks)?
- Yes
- No
  - 1. If yes, who has been involved in the last update of your local guideline? *(multiple answers possible)*
- Neonatologist
- Neonatologist with hemodynamic skills (hemodynamic consultant)
- Pediatric cardiologist
- Others, namely *(please specify)*

## Screening strategy

- 1. Are preterm infants routinely screened echocardiographically for (hemodynamic significant) PDA?
- Yes
- No, only if indicated by attending neonatologist
  - 1. If yes, when is this echocardiographic screening scheduled?
- Within 24 hours postnatal age (PNA)
- Between 24-72 hours PNA
- At the end of the first week
- Other *(please specify)*
  - 1. If yes, which infants are routinely screened?
- Based on gestational age only *(please specify)*
- Based on birth weight only *(please specify)*
- Based on both gestational age and/or birthweight *(please specify)*
- Other *(please specify)*
  - 1. If no, what would be reason(s) to perform echocardiography to evaluate a potential transductal shunt in patient who are not routinely screened? *(multiple answers possible)*
- Based on clinical signs
- Based on additional assessments
  - - 1. If based on clinical signs, please specify clinical signs *(multiple answers possible)*
- Wide pulse pressure
- Heart murmur
- Hyperactive precordium
- Systemic hypotension (low MAP)
- Low diastolic arterial pressure
- Inotropic support
- Bounding pulses
- Tachypnea / pulmonary edema
- Increased oxygen requirement
- Extubation failure
- CPAP failure
- Ventilator dependency
- Feeding intolerance
- Renal impairment
  - - 1. If based on additional assessments, please specify additional assessments *(multiple answers possible)*
- Chest radiograph
- NIRS monitoring
- Cerebral ultrasound with absent or reversed diastolic flow on Doppler imaging
- Signs of renal failure (increased creatinine level; oliguria)
- NT-pro-BNP
- Blood lactate concentration
  1. Would you perform an echocardiography to assess a PDA before a second dose of surfactant?
- Yes
- No
  - 1. If yes, would you administer surfactant to a patient with a large left to right transductal shunt volume?
- Yes
- No

## Diagnostic criteria for (hs)PDA

- 1. Who performs echocardiography to assess ductal patency? *(multiple answers possible)*
- Neonatologist (Neonatologist Performed Echocardiography – NPE)
- Neonatologist hemodynamic consultant (Targeted Neonatal Echocardiography - TNE)
- Neonatologist (Certificate in Clinician Performed Ultrasound - CCPU)
- Pediatric Cardiologist
- Neonatologist AND pediatric cardiologist
- Neonatologist OR pediatric cardiologist
  1. Are pediatric cardiologists involved to exclude congenital heart defects?
- No, echocardiographic assessment is performed by a neonatologist with focus on PDA only
- No, echocardiographic assessment is performed by a neonatologist with expertise to confirm structural normality of the heart
- Yes, echocardiographic assessment is performed by neonatologist and reviewed by a pediatric cardiologist
- Yes, initial echocardiographic assessment is always performed by a pediatric cardiologist to exclude congenital heart defects
  1. Which echocardiographic parameter(s) are used in your center to assess hemodynamic significance (shunt volume) of a persistent PDA *(multiple answers possible – rank the chosen parameters based on priority)?*
- PDA characteristics
  - Ductal diameter
  - Transductal flow direction
  - Transductal flow pattern (growing, pulsatile (non-restrictive) or restrictive pattern)
- Indices of pulmonary overcirculation
  - LVO - Left ventricular output
  - LVO:SVC ratio (SVC – superior vena cava flow)
  - LA:Ao ratio
  - LVEDD – Left ventricular end-diastolic dimension (Z-score)
  - Pulmonary vein d wave velocity
  - LPA diastolic velocity
  - Mitral valve E:A ratio
  - IVRT – isovolumic relaxation time
- Indices of systemic hypoperfusion
  - Abnormal OR retrograde diastolic flow (‘ductal steal’) in middle cerebral OR pericallosal artery
  - Abnormal OR retrograde diastolic flow (‘ductal steal’) in superior mesenteric artery
  - Abnormal OR retrograde diastolic flow (‘ductal steal’) in celiac trunk
  - Abnormal OR retrograde diastolic flow (‘ductal steal’) in descending aorta
- Other echocardiographic measurements *(please specify)*
  1. Which of the following variables do you use to determine hemodynamic significance of a persistent PDA? *(multiple answers possible)*
- Clinical parameters
  - Wide pulse pressure
  - Heart murmur
  - Hyperactive precordium
  - Systemic hypotension
  - Low diastolic blood pressure
  - Inotropic support
  - Bounding pulses
  - Oliguria
  - Tachypnea / pulmonary edema /hemorrhage
  - Increased oxygen requirement
  - Ventilator dependency
  - Feeding intolerance
  - Metabolic acidosis
- Need for respiratory support
  - nCPAP
  - nIPPV
  - Invasive ventilation
- Chest X-ray
- Echocardiography *(please specify cut-off values for chosen variables for small and large shunt)*
  - PDA diameter
    1. Used parameter for PDA diameter
- mm
- mm/kg
- PDA:LPA
  - Transductal flow velocity – v_max_
  - Transductal flow pattern
    1. I consider a growing pattern as a sign of a
- Small shunt
- Moderate shunt
- Large shunt
  - 1. I consider a pulsatile (non-restrictive) pattern as a sign of a
- Small shunt
- Moderate shunt
- Large shunt
  - 1. I consider a restrictive pattern as a sign of a
- Small shunt
- Moderate shunt
- Large shunt
  - LVO – Left ventricular output
  - LVO:SVC ratio (SVC – superior vena cava flow)
  - LA:Ao ratio
  - LVEDD – Left ventricular end-diastolic dimension
  - Pulmonary vein d wave velocity
  - LPA diastolic velocity
  - Mitral valve E:A ratio
  - IVRT – isovolumic relaxation time
- Biomarkers *(please specify)*
- NIRS *(please specify)*
- Other *(please specify)*
  1. Do you use a PDA severity or staging score?
- PDA severity score, as defined by El-Khuffash et al.[9]
- PDA staging system, as defined by McNamara and Sehgal[15]
- Other *(please specify)*
- No, no scoring system used

## Treatment strategy

- 1. What is your approach to early fluid management in preterm infants (< 28 weeks)?
- Restrictive daily fluid intake
- Normal daily fluid intake
- Increase daily fluid intake
  1. What is the preferred timing of PDA treatment in your center?
- Prophylaxis (< 24 hours postnatal age – PNA) WITHOUT echocardiogram
- Echocardiography guided targeted prophylaxis (< 24 hours PNA)
- Early targeted treatment based on screening echo (PNA 24-72 hours)
- Symptomatic treatment (PNA > 72 hours)
- Expectant treatment(s)
  1. What is the lower limit of your p_a_CO_2_ target range in preterm infants (< 28 weeks)? *(please specify)*
  2. What is the upper limit of your p_a_CO_2_ target range in preterm infants (< 28 weeks)? *(please specify)*
  3. What is the lower limit of your hematocrit target range in preterm infants (< 28 weeks)? *(please specify)*
  4. What is the upper limit of your hematocrit target range in preterm infants (< 28 weeks)? *(please specify)*
  5. What is the lower limit of your (transcutaneous) oxygen saturation target range in preterm infants (< 28 weeks)? *(please specify)*
  6. What is the upper limit of your (transcutaneous) oxygen saturation target range in preterm infants (< 28 weeks)? *(please specify)*
  7. Who decides to start (non-prophylactic) treatment?
- Neonatologist
- Neonatologist hemodynamic consultant
- Pediatric cardiologist
- Consensus between pediatric cardiologist AND neonatologist
- Pediatric cardiologist OR neonatologist
  1. What is the drug of first choice in your center?
- Ibuprofen
- Indomethacin
- Paracetamol/acetaminophen
  - 1. Do you start with a loading dose?
- Yes *(please specify loading and subsequent dose(s))*
- No *(please specify dosage)*
  - 1. What is te preferred route of administration?
- p.o.
- i.v.
  - 1. What is the total number of doses (including eventual loading dose)? *(please specify)*
  1. After failure of the initial drug course, which of the following interventions apply *(multiple answers possible)?*
- None
- Switch to another drug
- Surgical ligation
- Catheter closure
- Second course
  1. What is the drug of first choice in your center?
- Ibuprofen
- Indomethacin
- Paracetamol/acetaminophen
  - 1. Do you start with a loading dose?
- Yes *(please specify loading and subsequent dose(s))*
- No
  - 1. What is te preferred route of administration?
- p.o.
- i.v.
  - 1. What is the total number of doses (including eventual loading dose)? *(please specify)*
  1. When do you consider ductal ligation or catheter closure (either in your own hospital or referral of patients to an affiliating cardiothoracic center)?
- As primary treatment for preterm infants with a contraindication to pharmacological treatment (i.e. IVH, NEC)
- After failed medical treatment, if still hemodynamically significant PDA (any definition), same criteria as for medical treatment
- After failed medical treatment, if still hemodynamically significant PDA (any definition), stricter criteria than for medical treatment
- No patient receives ductal ligation, nor is transferred to an expert center
  1. How many courses of Ibuprofen, Indomethacin or Paracetamol do you tend to give before considering surgical or transcatheter PDA closure?
- One
- Two
- Three
- Four
- Other *(please specify)*
  1. Do you adjust the dosage when postnatal age increases?
- Yes *(please specify)*
- No
  1. Do you consider transcatheter closure an option for patients with a persistent PDA?
- Yes
- No
  1. Is transcatheter closure performed in your center?
- Yes
- No
  1. What is your approach towards enteral feeding during pharmaceutical PDA treatment?
- Stop enteral feeding
- No increase of enteral feeding during the course
- Normal advance of enteral feeding during the course
  1. How would you treat systemic hypotension in a patient with echocardiographic confirmed high transductal left to right shunt?
- Transductal flow modulation (i.e. by increasing the hematocrit, mean airway pressure or p_a_CO_2_)
- Volume expansion
- Dopamine
- Dobutamine
- Other *(please specify)*
  1. If next to transductal left to right shunting an initial short period of right to left shunting is observed (< 30% of heart cycle), what would you do?
- I would never treat the PDA
- I would only treat the duct if other indicators of significant LtR transductal shunt volume are present
- This does not influence my decision whether to treat or not

## Treatment efficacy metrics

- 1. After initiation of active treatment, how is the effect monitored and evaluated?
- Echocardiography after each dose and possible limitation of the total number of doses
- Echocardiography after a full course
- Clinical follow-up
- Other *(please specify)*
  1. Which effect do you consider as a therapeutic success and therefore stop further active treatment?
- Complete ductal closure
- From hemodynamic significant to non-hemodynamic significant PDA
- Clinical improvement
- Clinical improvement and non-hemodynamic significant PDA
  1. How do you manage an undocumented DA closure at discharge?
- Outpatient echocardiographic follow-up
- Outpatient clinic follow up with only physical examination (i.e. follow-up of the murmur and possible clinical signs)
- No (standard) follow-up
  1. Would there be a difference in your answers when focusing only on extreme preterm infants < 24 weeks (instead of < 28 weeks)?
- Yes *(please specify)*
- No

## Clinical equipoise – statements

- 1. PDA should be considered as an epiphenomenon of prematurity (an indicator of immaturity) rather than a leading cause of mortality and morbidity (scale 1-10, with 1 totally disagree and 10 totally agree)
     1. Therefor it should not be treated at all (scale 1-10)
     2. Therefor screening for ductal patency is not indicated at all (scale 1-10)
  2. PDA should be considered as an important cause of mortality and morbidity in preterm infants (scale 1-10)
     1. Therefor screening for ductal patency is essential (scale 1-10)
     2. Therefor it should be treated aggressively (scale 1-10)
     3. Therefor it should be treated early (scale 1-10)
  3. The PDA diameter is a good surrogate for shunt volume (scale 1-10)
  4. I would only consider treating a PDA in case of ‘hemodynamic significance’ (any definition) (scale 1-10)
  5. I would consider treating a ‘non-hemodynamic significant’ (any definition) PDA in case of associated clinical findings / morbidity (scale 1-10)
