# Supplementary material for: Survey highlighting the lack of consensus on diagnosis and treatment of patent ductus arteriosus in prematurity
Source: Eur J Pediatr. 2022 Mar 19;181(6):2459–68. doi: 10.1007/s00431-022-04441-8 (PMC9110525; doi:10.1007/s00431-022-04441-8)
Supplement: Supplementary file 2 — Supplementary file2 (DOCX 20 KB) [file 431_2022_4441_MOESM2_ESM.docx]

**Supplement 2 –** Checklist for Reporting Results of Internet E-Surveys (CHERRIES)[[13](file:///C:\Users\z378243\AppData\Local\Microsoft\Windows\Temporary%20Internet%20Files\Content.Outlook\HMMOAO9U\2021-11-30%20Eur%20J%20Ped%20EJPE-D-21-00593%20(tracked%20changes)_WPdB_TH_WPdB.docx#_ENREF_13)]

| Design | | |
| --- | --- | --- |
|  | Describe survey design | The target population was mainly neonatologists with interest in neonatal hemodynamics in general and/or patent ductus arteriosus specifically. To get a sample frame members of the European Society for Pediatric Research (ESPR) section Circulation, Oxygen Transport and Hematology and principal investigators of our BeNeDuctus trial were invited by email. Other respondents were recruited via the ESPR newsletter and Joint European Neonatal Society conference 2019. |
| Institutional Review Board approval and informed consent process | | |
|  | Institutional review board approval | A waiver has been obtained for this study from the Ethical committee at the Radboud university medical center (#2021-7469). |
|  | Informed consent | Potential participants were contacted by email and informed about the length and purpose of the survey and that data was collected and analyzed anonymously. |
|  | Data protection | No personal information was collected or stored. |
| Development and pretesting | | |
|  | Development and testing | The survey was originally designed by two authors (TH and WdB) based on the available literature and build in Castor®. The survey was tested and then reviewed and edited by the other authors (AK and PM). The final survey showed an intra-observer reliability of 97% in two participants. |
| Recruitment process and description of the sample having access to the questionnaire | | |
|  | Open survey versus closed survey | Closed survey. |
|  | Contact mode | Potential participants were contacted by mail and allowed or Web-based data entry. |
|  | Advertising the survey | The survey was advertised in the ESPR newsletter, on the Joint European Neonatal Societies conference in Maastricht 2019 and distributed to the mailing list of the ESPR Section Circulation, Oxygen Transport and Hematology and the principal investigators of the BeNeDuctus trial. |
| Survey administration | | |
|  | Web/E-mail | Web-based survey for which participants were contacted by email with a personal link to Castor®. In Castor® data was filled in by the participants and could be extracted from the Castor® database. |
|  | Context | Members of the ESPR section Circulation, Oxygen Transport and Hematology are neonatologist and trainees with special interest in neonatal hemodynamics in general. Most of them are also (clinical) researchers, although not all on the patent ductus arteriosus. ESPR newsletter recipients are pediatric researchers and clinicians or trainees, not specifically neonatologist nor neonatal hemodynamic interested and comparable to the Joint European Neonatal Society congress visitors, of which ESPR is one of the organizing societies.  The survey was therefore mainly filled in by neonatologist, half of them published on the PDA. By inviting the special interest group one might induce selection bias, which was tried to be reduced by also spreading the survey on the other, more general neonatologists, platforms. On the other hand it was one of our goals to gain insight in the presence or absence of heterogeneity within this special interest group on PDA diagnoses and management. |
|  | Mandatory/voluntary | Voluntary survey. |
|  | Incentives | No incentives were offered. |
|  | Time/Date | The survey was sent out between September 2019 and March 2020. |
|  | Randomization of items or questionnaires | Not performed. |
|  | Adaptive questioning | Certain items were only conditionally displayed based on responses to other items, for example the dosage of ibuprofen was only questioned when ibuprofen was chosen as drug of choice. |
|  | Number of Items | The number of items per page differed between the sections of the questionnaire, mainly between 10 to 20 items, as is shown in Supplement 1. |
|  | Number of screens (pages) | Each section, as shown in Supplement 1, had its own screen, so in total 7 screens. |
|  | Completeness check | Completeness check was done, participants could not proceed to the next page/section in Castor® if multiple choice questions had not been answered. Open fields could be filled in with a prespecified range to reduce the number of incorrect values. |
|  | Review step | Participants could navigate back in the survey and change responses as at the end of each page one could go back or proceed to the next part of the survey. |
| Response rates | | |
|  | Unique site visitor | Not applicable. |
|  | View rate (Ratio of unique survey visitors/unique site visitors) | Not applicable. |
|  | Participation rate (Ratio of unique visitors who agreed to participate/unique first survey page visitors) | Filled in part of the survey/Invited participants via mail = 71/144 = 49% |
|  | Completion rate (Ratio of users who finished the survey/users who agreed to participate) | Completed the survey/Filled in part of the survey = 56/71 = 79% |
| Preventing multiple entries from the same individual | | |
|  | Cookies used | Not applicable. |
|  | IP check | Not applicable. |
|  | Log file analysis | Not applicable. |
|  | Registration | All participants received a unique link which could be completed once. Incomplete surveys could be assessed and completed on a later moment. |
| Analysis | | |
|  | Handling of incomplete questionnaires | Incomplete questionnaires were also analyzed if baseline characteristics and at least one section (page) was completed. |
|  | Questionnaires submitted with an atypical timestamp | Not applicable. |
|  | Statistical correction | No statistical corrections were performed. |
