# Supplementary material for: Survey highlighting the lack of consensus on diagnosis and treatment of patent ductus arteriosus in prematurity
Source: Eur J Pediatr. 2022 Mar 19;181(6):2459–68. doi: 10.1007/s00431-022-04441-8 (PMC9110525; doi:10.1007/s00431-022-04441-8)
Supplement: Supplementary file 3 — Supplementary file3 (DOCX 28 KB) [file 431_2022_4441_MOESM3_ESM.docx]

**Supplement 3 –** Responses to survey

**Table S1 –** Baseline characteristics for all respondents (n=71)

| **Question** | | **n** |
| --- | --- | --- |
| **1.1. I am a** | |  |
| neonatologist | | 65 (92) |
| neonatal fellow | | 2 (3) |
| pediatric cardiologist | | 2 (3) |
| other, namely *pediatrician* | | 2 (3) |
| **1.2. I have been working on a neonatal intensive care unit for** | |  |
| < 10 years | | 20 (28) |
| 10-20 years | | 30 (42) |
| > 20 years | | 21 (30) |
| **1.3. I am currently working in (see also Figure S1)** | |  |
| Australia | | 3 (4) |
| Austria | | 1 (1) |
| Belgium | | 6 (8) |
| Brazil | | 2 (3) |
| Canada | | 5 (7) |
| Chile | | 3 (4) |
| Denmark | | 3 (4) |
| England | | 1 (1) |
| France | | 1 (1) |
| Germany | | 3 (4) |
| Greece | | 1 (1) |
| Ireland | | 3 (4) |
| Israel | | 2 (3) |
| Italy | | 2 (3) |
| Mexico | | 1 (1) |
| Norway | | 1 (1) |
| Oman | | 1 (1) |
| Poland | | 1 (1) |
| Romania | | 1 (1) |
| South Africa | | 5 (7) |
| Spain | | 2 (3) |
| Switzerland | | 1 (1) |
| The Netherlands | | 15 (21) |
| Turkey | | 2 (3) |
| USA | | 5 (7) |
| **1.4. I work in a …, as defined by American Academy of Pediatrics Committee on Fetus And Newborn[14]** | |  |
| Level II neonatal care unit | | 3 (4) |
| Level III neonatal care unit | | 23 (32) |
| Level IV neonatal care unit | | 45 (63) |
| **1.5. Have you been funded to investigate on the PDA?** | |  |
| Yes | | 16 (23) |
| No | | 55 (77) |
| **1.6. Have you published on the PDA? If yes, how many publications did you (co-)author?** | |  |
| Yes | 1-5 papers | 29 (41) |
|  | 5-10 papers | 4 (6) |
|  | > 10 papers | 3 (4) |
| No | | 35 (49) |
| **1.7. Yearly admissions of preterm infants (24-28 weeks)** | |  |
| < 25 preterm infants 24-28 weeks | | 10 (14) |
| 25-50 preterm infants 24-28 weeks | | 27 (38) |
| 50-100 preterm infants 24-28 weeks | | 25 (35) |
| > 100 preterm infants 24-28 weeks | | 9 (13) |
| **1.8. Yearly admissions of extreme preterm infants < 24 weeks** | |  |
| < 10 extreme preterm infants < 24 weeks | | 47 (66) |
| 10-25 extreme preterm infants < 24 weeks | | 22 (31) |
| 25-50 extreme preterm infants < 24 weeks | | 2 (3) |
| **1.9. Is there a pediatric cardiology service available at your institution?** | |  |
| Yes, 24/7 | | 52 (73) |
| Yes, but only during daytime | | 11 (15) |
| No, only remote consultation | | 6 (8) |
| No | | 2 (3) |
| **1.10. Do patients have to be transferred to another center for surgical ductal ligation? If no, where is surgical ligation (mostly) performed?** | |  |
| Yes | | 22 (31) |
| No | In the NICU | 35 (49) |
|  | In the operating room (OR) | 12 (17) |
|  | Cath lab | 2 (3) |

Data are presented as number (percentage). Percentages may not sum to 100 due to rounding.

**Table S2 –** Guidelines

| **Question** | **n** |
| --- | --- |
| **2.1. Is there a national guideline available regarding the diagnosis and management of persistent PDA in preterm infants (< 28 weeks)? (n=70)** |  |
| Yes | 4 (6) |
| No | 66 (94) |
| **2.2. Is there a local guideline available at your institution regarding the diagnosis and management of persistent PDA in preterm infants (< 28 weeks)? (n=71)** |  |
| Yes | 40 (56) |
| No | 31 (44) |
| **2.2.1 If yes, who has been involved in the last update of your local guideline? (n=40)** |  |
| Neonatologist with hemodynamic skills (hemodynamic consultant) | 30 |
| Neonatologist | 25 |
| Pediatric cardiologist | 20 |
| Others, namely *cardiac surgeon* | 2 |

Data are presented as number (percentage) for multiple-choice questions with single-answer possibility and number for multiple-answer possibilities. Percentages may not sum to 100 due to rounding.

**Table S3 –** Screening strategy

| **Question** | | **N** |
| --- | --- | --- |
| **3.1. Are preterm infants routinely screened echocardiographically for (hemodynamic significant) PDA? (n=71)** | |  |
| Yes | | 33 (46) |
| No | | 38 (54) |
| **3.1.1. If yes, when is this echocardiographic screening scheduled? (n=33)** | |  |
| Within 24 hours postnatal age (PNA) | | 7 (21) |
| Between 24-72 hours PNA | | 20 (61) |
| At the end of the first week | | 5 (15) |
| Other *variably typically within the first week* | | 1 (3) |
| **3.1.2. If yes, which infants are routinely screened? (n=31)** | |  |
| Based on gestational age only, *namely* | *Gestational age < 26-28 weeks* | 10 (32) |
|  | *Gestational age < 30-32 weeks* | 4 (13) |
| Based on both gestational age and/or birthweight*, namely* | *GA < 28 weeks AND/OR BW < 1000 gram* | 7 (23) |
|  | *GA < 30 weeks AND/OR BW < 1500 gram* | 5 (16) |
|  | *GA < 32 weeks AND/OR BW < 1500 gram* | 5 (16) |
| **3.2. Would you perform an echocardiography to assess a PDA before a second dose of surfactant? (n=71)** | |  |
| Yes | | 15 (21) |
| No | | 56 (79) |
| **3.2.1. If yes, would you administer surfactant to a patient with a large left to right transductal shunt volume? (n=15)** | |  |
| Yes | | 2 (13) |
| No | | 13 (87) |

Data are presented as number (percentage) for multiple-choice questions with single-answer possibility and number for multiple-answer possibilities. Percentages may not sum to 100 due to rounding.

**Table S4 –** Diagnostic criteria for (hs)PDA

| **4.1 Who performs echocardiography to assess ductal patency? (n=71)** | | n |
| --- | --- | --- |
| Pediatric cardiologist | | 47 |
| Neonatologist Performed Echocardiography (NPE) AND/OR Targeted Neonatal Echocardiography (TNE) AND/OR Certificate in Clinician Performed Ultrasound (CCPU) | | 40 |
| Neonatologist AND pediatric cardiologist | | 22 |
| Neonatologist OR pediatric cardiologist | | 16 |
| **4.2 Are pediatric cardiologists involved to exclude congenital heart defects? (n=71)** | |  |
| Yes, initial echocardiographic assessment is always performed by a pediatric cardiologist to exclude congenital heart defects | | 40 (56) |
| Yes, echocardiographic assessment is performed by a neonatologist and reviewed by a pediatric cardiologist | | 19 (27) |
| No, echocardiographic assessment is performed by a neonatologist with expertise to confirm structural normality of the heart | | 12 (17) |
| **4.3 Which echocardiographic parameter(s) are used in your center to assess hemodynamic significance (shunt volume) of a persistent PDA? (n=71)** | |  |
| *PDA characteristics* | | 70 |
| Ductal diameter | | 65 |
| Transductal flow pattern (growing, pulsatile (non-restrictive) or restrictive pattern) | | 63 |
| Transductal flow direction | | 58 |
| *Indices of pulmonary overcirculation* | | 53 |
| LA:Ao ratio | | 47 |
| LPA diastolic velocity | | 30 |
| LVO - Left ventricular output | | 27 |
| LVEDD – Left ventricular end-diastolic dimension (Z-score) | | 21 |
| Mitral valve E:A ratio | | 20 |
| IVRT – isovolumic relaxation time | | 13 |
| Pulmonary vein d wave velocity | | 11 |
| LVO:SVC ratio (SVC – superior vena cava flow) | | 7 |
| *Indices of systemic hypoperfusion* | | 52 |
| Abnormal OR retrograde diastolic flow (‘ductal steal’) in descending aorta | | 42 |
| Abnormal OR retrograde diastolic flow (‘ductal steal’) in middle cerebral OR pericallosal artery | | 33 |
| Abnormal OR retrograde diastolic flow (‘ductal steal’) in superior mesenteric artery | | 31 |
| Abnormal OR retrograde diastolic flow (‘ductal steal’) in celiac trunk | | 30 |
| **4.4 Which of the following variables do you use to determine hemodynamic significance of a persistent PDA? (n=71)** | |  |
| *Clinical parameters* | | 55 |
| Ventilator dependency | | 45 |
| Increased oxygen requirement | | 44 |
| Tachypnea / pulmonary edema / hemorrhage | | 43 |
| Metabolic acidosis | | 42 |
| Low diastolic blood pressure | | 39 |
| Wide pulse pressure | | 38 |
| Systemic hypotension (low mean arterial pressure) | | 38 |
| Inotropic support | | 38 |
| Oliguria | | 35 |
| Hyperactive precordium | | 27 |
| Feeding intolerance | | 27 |
| Heart murmur | | 26 |
| Bounding pulses | | 22 |
| *Need for respiratory support* | | 58 |
| Invasive ventilation | | 57 |
| Nippv | | 22 |
| nCPAP | | 20 |
| *Chest X-ray* | | 24 |
| *Echocardiography* | | 68 |
| PDA diameter | | 60 |
|  | [mm] | 43 |
|  | [mm/kg] | 9 |
|  | PDA:LPA | 5 |
| LA:Ao ratio | | 47 |
| Transductal flow pattern | | 44 |
| Transductal flow velocity (v_max_) [m/s] | | 28 |
| LVO – Left ventricular output [ml/kg/min] | | 25 |
| LPA diastolic velocity [m/s] | | 19 |
| LVEDD – Left ventricular end-diastolic dimension [mm] | | 17 |
| Mitral valve E:A ratio | | 15 |
| IVRT – isovolumic relaxation time [ms] | | 12 |
| LVO:SVC ratio (SVC – superior vena cava flow) | | 5 |
| Pulmonary vein d wave velocity [m/s] | | 4 |
| *Biomarkers* | | 9 |
| (NT-pro) BNP | | 3 |
| Lactate | | 2 |
| *NIRS* | | 14 |
| Cerebral | | 8 |
| Splanchnic/mesenterial | | 2 |
| **4.5 Do you use a PDA severity or staging score? (n=59)** | |  |
| No, no scoring system used | | 49 (83) |
| PDA staging system, as defined by McNamara and Sehgal[15] | | 7 (12) |
| PDA severity score, as defined by El-Khuffash et al.[9] | | 3 (5) |

Data are presented as number (percentage) for multiple-choice questions with single-answer possibility and number for multiple-answer possibilities. Percentages may not sum to 100 due to rounding.

**Table S5 –** Treatment strategy

| **5.1. What is your approach to early fluid management in preterm infants (< 28 weeks)? (n=62)** | | n |
| --- | --- | --- |
| Restrictive daily fluid intake | | 14 (23) |
| Normal daily fluid intake | | 45 (73) |
| Increase daily fluid intake | | 3 (5) |
| **5.2. What is the preferred timing of PDA treatment in your center? (n=61)** | |  |
| Prophylaxis (< 24 hours postnatal age – PNA) WITHOUT echocardiogram | | 1 (2) |
| Echocardiography guided targeted prophylaxis (< 24 hour PNA) | | 2 (3) |
| Early targeted treatment based on screening echo (PNA 24-72 hours) | | 26 (43) |
| Symptomatic treatment (PNA > 72 hours) | | 25 (41) |
| **5.3.-5.8 Target ranges in preterm infants (< 28 weeks) for** | LLN | ULN |
| p_a_CO_2_ [kPa] | 5.0 (4.6-6.0) | 8.0 (7.3-8.5) |
| Hematocrit [L/L] | 0.30 (0.29-0.35) | 0.63 (0.55-0.69) |
| (transcutaneous) oxygen saturation [%] | 90 (88-90) | 95 (94-95) |
| **5.9. Who decides to start (non-prophylactic) treatment? (n=59)** | |  |
| Neonatologist | | 35 (59) |
| Neonatologist hemodynamic consultant | | 8 (14) |
| Consensus between pediatric cardiologist AND neonatologist | | 14 (24) |
| Pediatric cardiologist OR neonatologist | | 2 (3) |
| **5.11. After failure of the initial drug course, which of the following interventions apply? (n=60)** | |  |
| Second course | | 50 |
| Switch to another drug | | 13 |
| None | | 4 |
| Surgical ligation | | 3 |
| Catheter closure | | 1 |
| **5.13. When do you consider ductal ligation or catheter closure (either in your own hospital or referral of patients to an affiliating cardiothoracic center) (n=58)** | |  |
| After failed medical treatment, if still hemodynamically significant PDA (any definition), stricter criteria than for medical treatment | | 39 (67) |
| After failed medical treatment, if still hemodynamically significant PDA (any definition), same criteria as for medical treatment | | 13 (22) |
| No patient receives ductal ligation, nor is transferred to an expert center | | 6 (10) |
| **5.14. How many courses of Ibuprofen, Indomethacin or Paracetamol do you tend to give before considering surgical or transcatheter PDA closure? (n=53)** | |  |
| Two | | 32 (60) |
| Three | | 17 (32) |
| Four | | 1 (2) |
| Other | | 3 (6) |
| **5.15. Do you adjust the dosage when postnatal age increases? (n=53)** | |  |
| Yes | | 19 (36) |
| No | | 34 (64) |
| **5.16. Do you consider transcatheter closure an option for patients with a persistent PDA? (n=54)** | |  |
| Yes | | 28 (52) |
| No | | 26 (48) |
| **5.17. Is transcatheter closure performed in your center? (n=54)** | |  |
| Yes | | 20 (37) |
| No | | 34 (63) |
| **5.18. What is your approach towards enteral feeding during pharmaceutical PDA treatment? (n=52)** | |  |
| Normal advance of enteral feeding during the course | | 31 (60) |
| No increase of enteral feeding during the course | | 15 (29) |
| Discontinuation of enteral feeding | | 6 (12) |
| **5.19. How would you treat systemic hypotension in a patient with echocardiographic confirmed high transductal left to right shunt? (n=54)** | |  |
| Dopamine | | 26 |
| Transductal flow modulation (i.e. by increasing the hematocrit, mean airway pressure or p_a_CO_2_) | | 23 |
| Dobutamine | | 12 |
| Other | | 12 |
| Volume expansion | | 8 |
| **5.20. If next to transductal left to right shunting an initial short period of right to left shunting is observed (<30% of heart cycle), what would you do? (n=71)** | |  |
| I would only treat the duct if other indicators of significant LtR transductal shunt volume are present | | 47 (66) |
| I would never treat the PDA | | 13 (18) |
| This does not influence my decision whether to treat or not | | 11 (15) |

Data are presented as number (percentage) for multiple-choice questions with single-answer possibility and number for multiple-answer possibilities. Percentages may not sum to 100 due to rounding. Median (interquartile range) for upper and lower limit of normal.
*LLN,* lower limit of normal; *ULN,* upper limit of normal.

**Table S6 –** Treatment efficacy metrics

| **6.1. After initiation of active treatment, how is the effect monitored and evaluated? (n=59)** | |  |
| --- | --- | --- |
| Echocardiography after a full course | | 43 (73) |
| Echocardiography after each dose and possible limitation of the total number of doses | | 10 (17) |
| Clinical follow-up | | 6 (10) |
| **6.2. Which effect do you consider as a therapeutic success and therefore stop further active treatment? (n=58)** | |  |
| From hemodynamic significant to non-hemodynamic significant PDA | | 25 (43) |
| Clinical improvement and non-hemodynamic significant PDA | | 16 (28) |
| Clinical improvement | | 9 (16) |
| Complete ductal closure | | 8 (14) |
| **6.3. How do you manage an undocumented DA closure at discharge? (n=59)** | |  |
| Outpatient echocardiographic follow-up | | 41 (69) |
| Outpatient clinic follow up with only physical examination (i.e. follow-up of the murmur and possible clinical signs) | | 11 (19) |
| No (standard) follow-up | | 7 (12) |
| **6.4. Would there be a difference in your answers when focusing only on extreme preterm infants < 24 weeks (instead of < 28 weeks)? (n=59)** | |  |
| Yes, *namely* | | 27 (46) |
|  | *More aggressive* | 21 |
|  | *Less aggressive / watchful waiting* | 2 |
| No | | 32 (54) |

Data are presented as number (percentage) for multiple-choice questions with single-answer possibility and number for multiple-answer possibilities. Percentages may not sum to 100 due to rounding
