# Supplementary figures and images for: Survey highlighting the lack of consensus on diagnosis and treatment of patent ductus arteriosus in prematurity
Source: Eur J Pediatr. 2022 Mar 19;181(6):2459–68. doi: 10.1007/s00431-022-04441-8 (PMC9110525; doi:10.1007/s00431-022-04441-8)

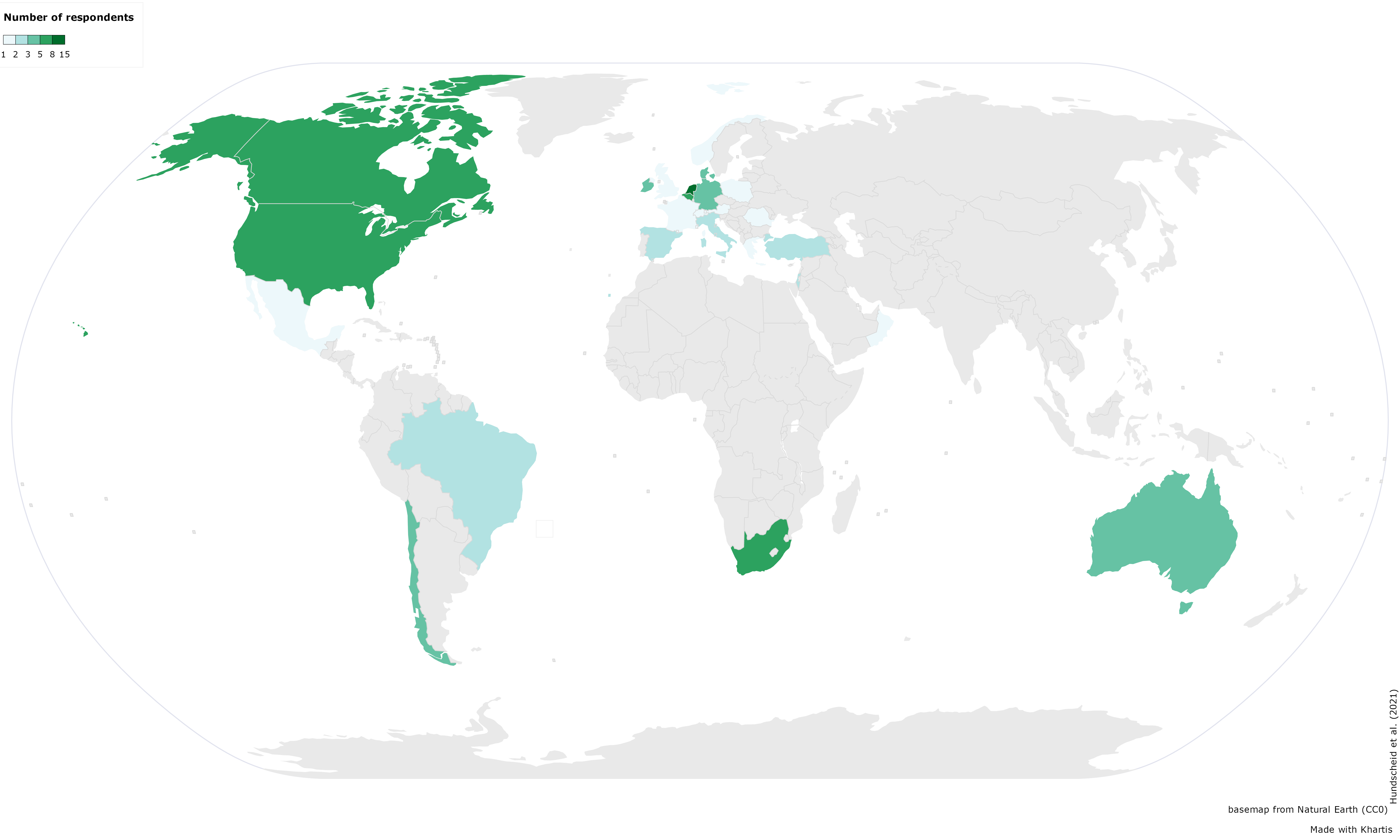

Supplement: Supplementary file 4 — Supplementary file4 (PNG 836 KB) [file 431_2022_4441_MOESM4_ESM.png]
